# Development of Marine Antifouling Epoxy Coating Enhanced with Clay Nanotubes

**DOI:** 10.3390/ma12244195

**Published:** 2019-12-13

**Authors:** Ye Fu, Wencai Wang, Liqun Zhang, Vladimir Vinokurov, Anna Stavitskaya, Yuri Lvov

**Affiliations:** 1 School of Materials Science and Mechanical Engineering, Beijing Technology and Business University, Beijing 100048, China; fuye@btbu.edu.cn; 2 State Key Laboratory of Organic-Inorganic Composites, Beijing University of Chemical Technology, Beijing 100029, China; 3 Department of Physical and Colloid Chemistry, Gubkin University, Moscow 119991, Russia; vladimir@vinokurov.me (V.V.); stavitsko@mail.ru (A.S.); 4 Institute for Micromanufacturing, Louisiana Tech University, Ruston 71272, LA, USA

**Keywords:** clay nanotubes, antifoulant encapsulation, sustained protection

## Abstract

An antifouling epoxy resin doped with natural clay nanotubes that are loaded with biocide or silver allowed extended protection against the proliferation of marine microorganisms. Compared to the 2–3 months of protection with antifoulant dichlorooctylisothiazolone (DCOIT) directly admixed into epoxy resin, the DCOIT release time of the halloysite formulations was extended to 12 months by incorporating biocide-loaded nanoclay in the polymer matrix. The protective properties of the epoxy-halloysite nanocomposites showed much less adhesion and proliferation of marine bacteria *Vibrio natriegens* on the resin surface after a two-month exposure to seawater than the coating formulations directly doped with non-encapsulated DCOIT. The coating formulation protection efficiency was further confirmed by twelve-month shallow field tests in the South China Sea. Replacing 2 wt.% biocide in the traditional formula with DCOIT-loaded natural environmentally friendly halloysite clay drastically improved the antifouling properties of the epoxy coating, promising scalable applications in protective marine coating. The antifouling property of epoxy resin was enhanced with silver particles synthesized on halloysite nanotubes. A natural mixture of MnO particles and halloysite could also be used as a nonbiocide additive to marine coating. The short-term White Sea water test of epoxy coating with 5% of Ag-halloysite composite of MnO-halloysite natural mixture showed no visible fouling.

## 1. Introduction

Biofouling starts with the accumulation of microorganisms, algae, and barnacles on vessels and equipment that are exposed to seawater, leading to higher frictional resistance, increadsed fuel consumption for ships, and shortened service life due to stimulated biocorrosion [1]. In addition, it shortens the intervals of ships dry-docking for fouling removal and repainting [2]. The higher fuel consumption results in an aggravation of CO_2_ emissions and large economic losses. The annual global loss caused by marine biofouling was estimated to be $150 billion in 2016 [3,4]. The aquaculture industry, which in the USA is more than a $2 billion per year business, employing over 16,000 people, also requires non-toxic and long-lasting antifouling coatings. For example, for the netting used in fish pens, biofouling rapidly accumulates and fills in the open areas of nets within weeks that leads to multiple problems such as fish diseases: netpen liver disease, amoebic gill disease, and parasites.

Bacteria colonize manmade materials immersed in seawater within hours. These early small colonizers form a biofilm: an assemblage of attached cells referred to as “slime”. Attached cells divide, rapidly giving rise to colonies that eventually coalesce to form a compact biofilm, which may achieve 1 mm in thickness, and can be a base for seaweeds and shells [5].

Current antifouling paints utilize agents that are toxic to a variety of marine species. The tributyltin (TBT) additive, which is effective after being added directly to paint [6,7], was banned for its toxicity and persistence in the sea [8,9]. The most popular substitute has been copper oxide, which today can be found in over 400 commercial marine paints. Although not as toxic as TBT, copper oxide is also harmful. It is not accepted by many aquaculture farmers because of their crops’ sensitivity to it, in particular those raising shrimp and shellfish. The paint additives containing other metals (zinc, titanium oxide) are still unsafe, causing destruction of crabs and scallops, which endangers marine ecology [10,11,12]. Ship coatings containing copper and zinc are still the most usable due to the affordable cost. Overuse of metal-based coating for ships and desks leads to leaching of copper and zinc to water, thus increasing their concentration in marine species, especially close to harbors [13,14].

Toxic and non-toxic coatings control fouling in different ways: toxic paint layers do not become fouled until all the active agents have been leached out; nontoxic coatings, such as silicones, rely on the “slipperiness” of their surfaces to release the microorganisms after settlement [15]. Silicone polymers have shown a good fouling release capability, accompanied by a smooth surface structure and a low glass transition temperature [16]. Although sea organisms can attach to silicones, they can be removed easily by brushing or hydrodynamic self-cleaning [17]. An improvement in fouling release capability due to the inclusions of oils in coatings (such as silicone or mineral oils) was demonstrated, although it requires encapsulation. Therefore, micro/nano encapsulation results in the very slow release of fungicides and oils and is a goal of new environmentally friendly marine antifouling coating technologies. Polymer microcapsules have been used for encapsulation [18,19]. However, their too-slow release rate, especially for high-effective-concentration biocides, leads to untimely supplementation of the antifoulant and loss of the antifouling properties of the composite. The non-closed encapsulation structures give a more controllable release rate, as for porous microspheres, microtubes and layered double hydroxides [20,21]. The high fabrication cost and complicated production process of these containers restricts their extended application.

Halloysite clay is a natural mineral, of which thousands of tons are available at a low price. Tubular halloysite is rolled-up aluminosilicate layers with 50–60 nm outer diameter, 10–15 nm lumen and 0.5–1 μm length. It is similar to kaolinite in composition, with additional water between adjacent layers in the wall [22]. The negatively charged outer silica surface and positively charged inner alumina surface of halloysite allows for selective loading of chemicals [23,24]. These clay nanotubes were used as tiny containers for encapsulation and controlled release of many active agents (catalysts, antioxidants, flame-retardants, anticorrosion agents, drugs, and proteins) [25,26,27]. The release time can be extended from days and months to years if the loaded clay nanotubes are embedded into polymers.

We focused on antifouling agents encapsulated into clay nanotube, making an extended years-long sustained release, without a harmful bust at the beginning of exploitation. Silver nanoparticles loaded in halloysite nanotubes could also be another prospect, due to their antimicrobial activity while being non-harmful to higher organisms. Silver nanoparticles have been extensively studied due to their antiseptic properties and their inhibition of biofilms, and their clay encapsulation promises advantages in long-lasting protection [28,29]. Combination of silver nanoparticles with DCOIT loaded into an antifouling coating show synergetic effects against fouling [30]. Naturally excavated cheap halloysite nanotubes can be used as a container for DCOIT antifouling agent and serve as a template for Ag-particles formation preventing their undesirable aggregation [31,32,33,34].

In this work, we report antifouling formulations based on epoxy resin doped at 22 wt.% with halloysite clay nanotubes loaded fungicides. The time of release of DCOIT from halloysite in water, non-polar solvent, and from doped epoxy composites demonstrates an essential elongation. The release time of the clay-encapsulated DCOIT was extended to 12 months, allowing for long-lasting antifoulant action, which is much more than 2–3 months of protection when non-encapsulated agents were directly admixed into epoxy.

The adhesion and proliferation of *Vibrio natriegens* on the epoxy composite was monitored. *Vibrio natriegens,* a gram-negative bacterium widely distributed in seas, lakes, and rivers, forms a primary fouling layer (biofilm) on the surfaces exposed to marine environments. The inhibition ability of microbial adhesion was further measured with the antifouling field tests by immersing coated panel in warm seawater at the Sanya Bay marine station (China) and in cold Arctic sea (Russia). The extended biofouling protection with nanoclay encapsulation containing DCOIT and Ag confirms the strategy for non-toxic, durable and effective antifouling composites. This method may be used as long-lasting coating for ships and marine structure. It also has an advantage for antifouling protection in cold Arctic waters, which is becoming much needed with marine petroleum exploration shifting to northern seas.

## 2. Experimental

### 2.1. Materials and Reagents

**Figure 1:**
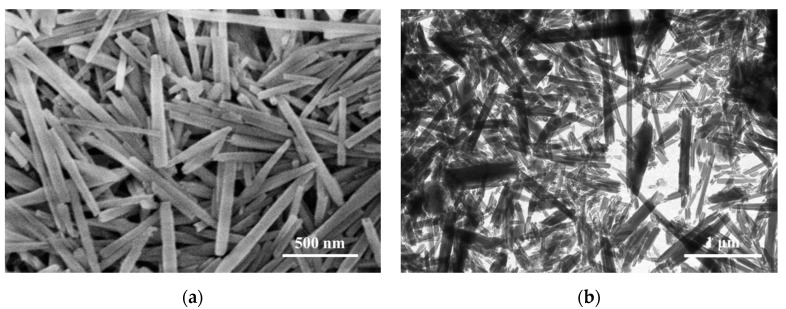
(**a**) SEM and (**b**) TEM images of the native halloysite nanotubes.

Halloysite (HNT) was purified and supplied by B. Zhang, Zhengzhou University, as described in [35]. The content of tubule halloysite clay is ca. 98%, and its image is presented in Figure 1. Fungicide 4,5-dichloro-2-octyl-isothiazolone (DCOIT) was purchased from Nantex Industry Co., Ltd., (Kaohsiung, China) and epoxy resin—from Tianjin Lions Paint Co., Ltd., (Tianjin, China). *Vibrio natriegens* ATCC 33899 was supplied by China General Microbiological Culture Collection Center (Beijing, China). Cyclohexane and ethanol were obtained from Beijing Chemical Plant (Beijing, China). All of these materials were used without further purification.

### 2.2. Preparation of DCOIT Loaded into Halloysite Nanotubes

Halloysite clay powder was dispersed in DCOIT-ethanol solution (50 mg·mL^−1^) and kept in a vacuum at 0.1 MPa for 10 min, followed by placement in atmospheric pressure; this cycle was repeated 3 times. After washing with water, halloysite was settled in a vacuum oven at 60 °C for 6 h. The dried halloysite nanotubes loaded with DCOIT (HNTs/DCOIT) were milled into a fine powder. The loading efficiency *C_D_* was calculated using Equation (1):

(1) $$C_{D}=\left(1-W_{H-D}/W_{H}\right)\times 100\%$$

*W_H-D_* is the weight of DCOIT-loaded halloysite at 600 °C, *W_H_* is the weight of pristine halloysite at 600 °C.

### 2.3. Antifouling Assay

The antifouling properties of epoxy resin composites with DCOIT were analyzed with monitoring of proliferation *Vibrio natriegens* ATCC 33899 as a model bacteria strain. *Vibrio natriegens* was transferred into 2216E liquid medium and shaken overnight at 30 °C. The epoxy resin sample of 10 × 10 × 2 mm^3^ was settled in the bacterial suspension (O.D._600 nm_ = 1.0) and incubated at 30 °C for 3 days. Three parallel tests were carried out for each sample. The unattached bacteria on the sample surface were removed by washing with the phosphate saline buffer. The fixation of bacteria was carried out with a conventional method by treating with 2% glutaraldehyde PBS solution for 4 h. The dehydration of bacteria was achieved by ethanol treatment at concentrations of 25, 50, 75, and 100%.

The field test of the epoxy resin composites with DCOIT was carried out in the Sanya Bay by China Shipbuilding Industry Corp. (Hainan, China) using the antifouling panels in shallow submergence method according to ASTM D 3623-78a (2012) [36]. Three panels with sizes of 350 × 250 mm^2^ coated with each epoxy resin formulation were soaked in seawater for one year.

### 2.4. Characterization

Scanning electron microscopy (SEM) images were taken with Hitachi S-4800 (Ibraraki, Japan) microscope at an accelerating voltage of 5 kV. Energy dispersive spectrometry (EDS) was performed on a HORIBA X-Max20 detector (Tokyo, Japan) attached to the SEM. Transmission electron microscopy (TEM) was performed with FEI Taicnai G2 20 STWIN (FEI, Hillsboro, OR, USA) at an accelerating voltage of 200 kV. Thermogravimetric analysis (TGA) of the samples was performed with TGA-60A (Shimadzu, Tokyo, Japan) in the temperature range from 30 to 600 °C at a heating rate of 10 °C·min^−1^ in a flow of nitrogen (50 mL·min^−1^). Fourier transform infrared spectroscopy (FTIR) was used to characterized pure DCOIT, pristine halloysite and antifoulant loaded inside the nanotubes with a TENSOR 27 (Bruker, Bremen, Germany) instrument after dispersing in KBr, followed by pressing these powders into pellets.

Ultraviolet-visible adsorption spectra (UV-vis, Agilent 8453, Agilent Technology, Palo Alto, CA, USA) were recorded for monitoring fungicide release (with stirring 50 mg of HNTs-DCOIT in 1 mL of water or cyclohexane; 2 g in 5 mL water halloysite-epoxy was used as a substitute). After centrifugation, the supernatant was taken for record and fresh solvent was added. The collected supernatant absorption peak at 283 nm was used to calculate concentration of released DCOIT.

## 3. Results and Discussion

### 3.1. Halloysite Lumens Encapsulating Antifoulant-DCOIT

**Scheme 1:**
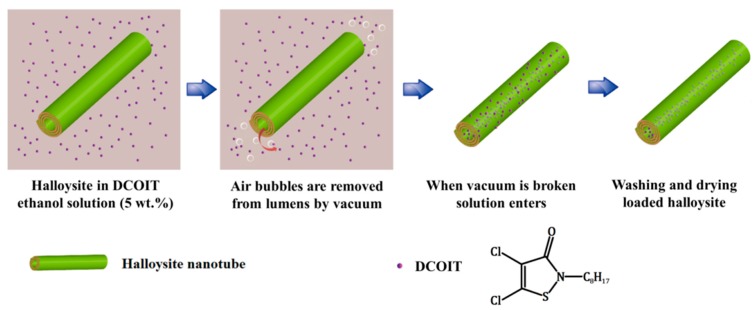
Loading of DCOIT into halloysite nanotubes.

As shown in Scheme 1, the inner cavity of halloysite was loaded by exposing the clay to concentrated DCOIT solution in ethanol. The air was removed from the lumens by vacuum. DCOIT solution entered into the nanotubes when the vacuum was broken.

**Figure 2:**
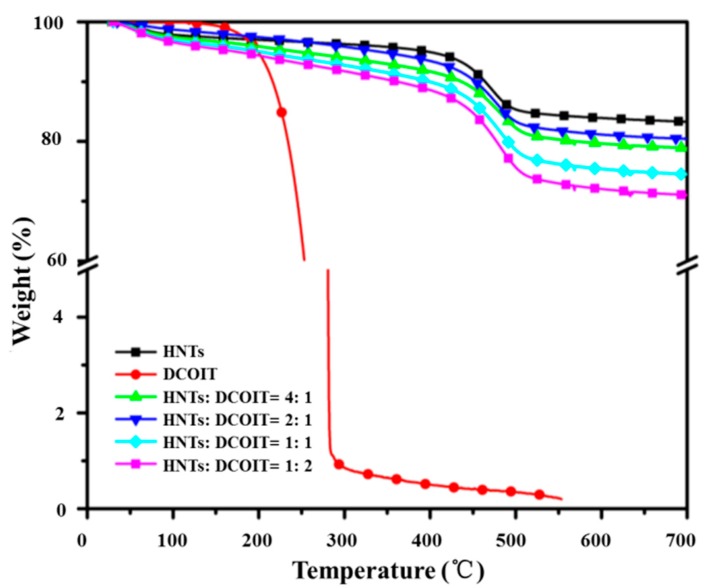
TGA curves of HNTs-DCOIT prepared at different weight ratios; the DCOIT concentration used for loading was 5 wt.%.

The loading efficiency of DCOIT prepared with different weight ratios was calculated with the temperature decomposition weight loss (Figure 2). It increased from 2.7 to a maximum of 12.0 ± 0.2 wt.% with higher DCOIT concentrations. Antifoulant encapsulation in halloysite nanotubes was optimized at 9–10 wt.%, which is close to the estimation of the nanotubes’ internal volume of ca. 10 vol.%. DCOIT condensation from a saturated solution could occur in the tube’s lumen during loading. Some extra DCOIT (taking into account a clay density of about double that of DCOIT) may be due to partial adsorption on the outer surface of the nanotubes and is released in the initial stage (before the release from the lumens). This may be efficient as the initiation for the first month bust-protection, allowing for a higher overall effect of the composite coating. The formulation of halloysite/inhibitor at a weight ratio of 1:1 with a loading efficiency of 9.0 ± 0.2 wt.% was used for further research.

**Figure 3:**
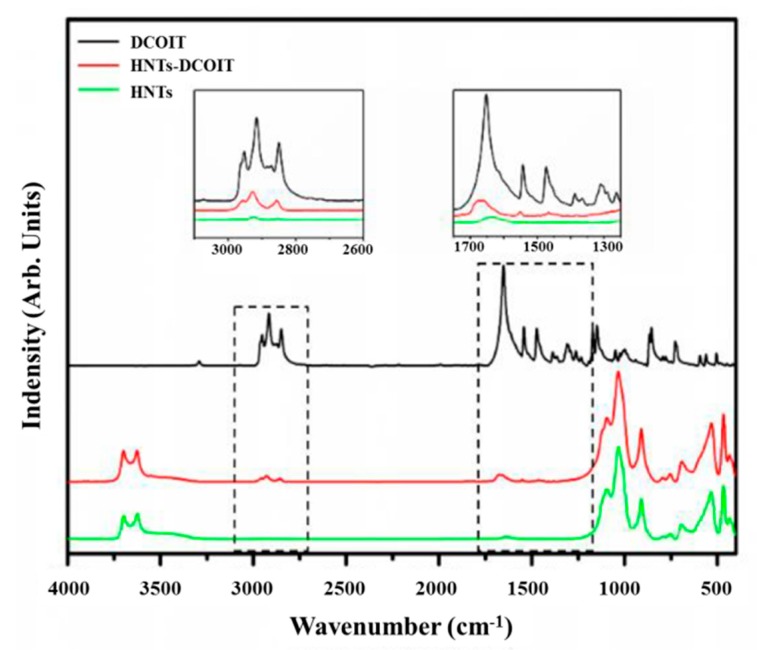
FT-IR spectra of antifoulant (DCOIT), halloysite (HNTs) and loaded halloysite (HNTs-DCOIT).

In the FT-IR spectrum (Figure 3), after encapsulating fungicide into halloysite, the vibrations of DCOIT molecules at 1723 cm^−1^ attributed to the C=O stretching, 1475 cm^−1^ due to the in-plane stretching of C=C, and 686 cm^−1^ related to the stretching of C–Cl were preserved. No new peaks appeared, which shows that DCOIT loading is a physical process (no chemical reactions occurred between the DCOIT and the nanoclay).

### 3.2. Release Kinetics of the Antifoulant

**Figure 4:**
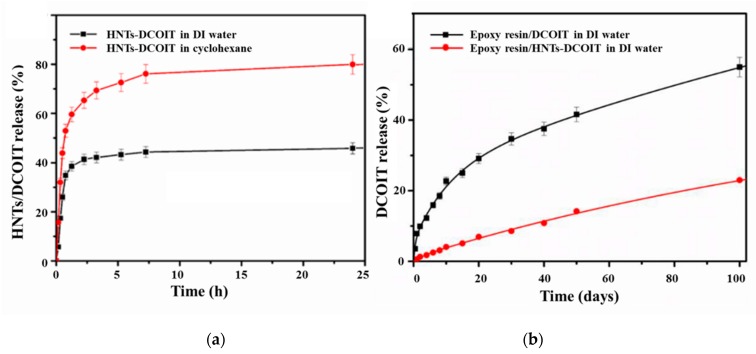
Antifoulant DCOIT release from (**a**) halloysite lumens in DI water and cyclohexane; (**b**) epoxy resin doped at 22 wt.% HNTs-DCOIT in water as compared with epoxy sample containing the same amount of non-encapsulated DCOIT (upper black curve).

UV-vis monitoring was used to study the kinetics of antifoulant release from the nanotubes, both in water and cyclohexane (as a model of organic non-polar media). It can be observed in Figure 4a that the release rate of DCOIT in water and cyclohexane is faster at the initial step, and then slows down before finally reaching a low increment region. The release amount and time at release equilibrium are different because of the different solubility of the antifoulant in cyclohexane and water. The release follows first-order exponential kinetics $M_{t}=M_{\infty }\times \left[1-e^{-\alpha t}\right]$, where $M_{\infty }$ is the amount of antifoulant released over an infinitely long time, and *α* is the release rate constant. In water, the release reached 44 ± 2 wt.% after 5 h. In cyclohexane, the release rate slowed down after 2 h and gradually reached 76 ± 2 wt.% in 7 h.

The release from the halloysite embedded in the epoxy resin matrix was drastically slowed down, reaching hundreds of days (Figure 4b). These release kinetics were recorded with two composite samples: the first, control sample was an epoxy resin admixed with pure non-encapsulated 7 wt.% antifoulant (epoxy/DCOIT), and the second, encapsulated formulation was an epoxy resin doped with 22 wt.% HNTs-DCOIT (halloysite loaded at 9.0 wt.% with DCOIT). Both composite formulations had equal 7 wt.% amounts of DCOIT.

Epoxy resin/HNTs-DCOIT enabled sustained biocide release from the nanotubes into the polymer matrix and its further migration to the surface led to the time-extended protection. The DCOIT release kinetic from halloysite-epoxy composite was described with Peppas’ model: $M_{t}=K\times t^{n}$, where *K* and *n* are experimental constants. Values of *K* decreased 16 times by composing halloysite encapsulated antifoulant into the resin matrix, while n increased from 0.4 to 0.8, becoming closer to the zero-order kinetics. This almost linear and slow release is a very favorable protection characteristic. After one month, the release of biocide in epoxy/HNTs-DCOIT was only 8.8 ± 0.5%, while that in non-encapsulation epoxy/DCOIT (direct fungicide admixing) 35.0 ± 2.0% of antifoulant were lost. The nanoclay encapsulation drastically slowed down the release of the antifoulant and prolonged the biocide’s supply for the coating protection. This leads to a year-long protection potential, as will be shown below for the test samples immersed in natural seawater.

### 3.3. Silver Nanoparticles on Halloysite and Its Epoxy Composites

Industry uses epoxy paints with the addition of silver nitrate and silver oxide as antimicrobial additives. A direct addition of the silver compounds to the paint has some disadvantages; in particular, paint acquires a characteristic yellow-brownish color due to the formation of larger silver particles. Additionally, silver nanoparticles absorb light and form plasmons, which accelerate polymer degradation. Suggested halloysite nanotube templated silver systems are better mixable with epoxy and have clay-attached Ag-nanoparticles, allowing for paint color preservation while possessing antibacterial properties. The incorporation of silver nanoparticles into the inner lumen of halloysite extends the silver stability and the lifetime of the antimicrobial effect, as shown earlier for dry polyurethane paint formulations not related to marine environments [37]. Antimicrobial activities of silver-halloysite composites depend on the amount of silver ions released: 10 mg/mL of halloysite-silver nanocomposites release ca. 1 mM of silver ions to the aqueous environment. This is 5 times more than for the silver oxide doped glass coating formulations and 20 times more than the corresponding data for silver doped carbon nanotubes, thus promising higher efficiency of halloysite antifouling formulations [38].

**Figure 5:**
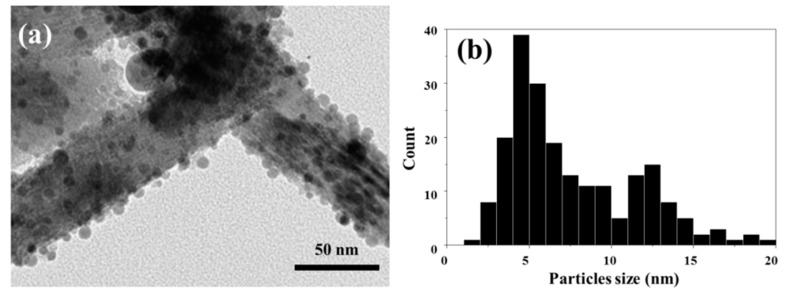
(**a**) TEM images and (**b**) particle size distributions of silver synthesized on the clay nanotubes.

We present here a method for silver-halloysite paint composite preparation for antifouling coatings with the ability to vary size of silver particles. It is based on optimization of silver ions adsorption from solution on the nanotubes and its reduction using different reduction agents. Figure 5 shows the tubule composite resulting from the reduction of silver ions adsorbed on APTES-modified halloysite using NaBH_4_ according to procedure modified from [39]. The produced silver particles have a bimodal distribution and are mostly 3 to 8 nm (72% particles) with a minor second peak at 12–14 nm. Reduction with NaBH_4_ produces Ag-particles not covered with stabilizers that may enhance Ag^+^ ions release to media.

In the second approach, we used tannic acid as a reduction agent and particle stabilizer: positively charged nanotubes modified with APTES were dispersed in solution and anionic tannic acid adsorbed on the surface due to electrostatic interaction. Silver nitrate solution was then added and a reduction of the Ag^+^ to Ag^0^ was achieved through the donation of electrons from tannic acid. Within 30 min, the formation of brownish silver nanoparticles was observed. Silver particles have an average diameter of 5 ± 1 nm with the prevalence of small particles. Halloysite nanotubes act as a clay template supporting silver particles preventing their aggregation.

**Figure 6:**
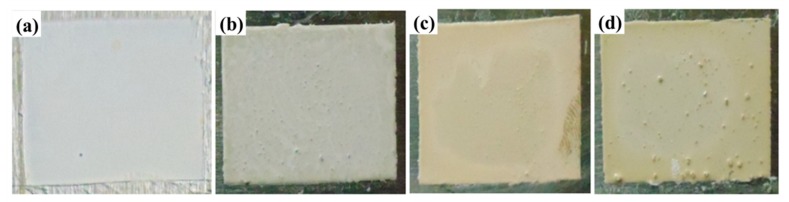
Images of epoxy paint after one-week sunlight exposure: (**a**) original paint, no silver added, (**b**) doped with 5 wt.% halloysite-silver composite, (**c**) with 1 wt.% non-encapsulated silver oxide nanoparticles and (**d**) with 1 wt.% non-encapsulated 20 nm silver nitrate particles.

To prevent coloring the epoxy, silver 4–5 nm particles were synthesized by selective placement of silver acetate inside halloysite nanotubes (followed by thermo-reduction). This technique enabled the preservation of a white epoxy paint color after doping with Ag-HNT; Figure 6. Such silver-clay core-shell tubule systems were added to epoxy paint at 5 wt.%, providing an efficient antimicrobial coating when tested with gram-negative *Escherichia coli* and gram-positive *Staphyloccocus aureus* bacteria [27]. Silver loaded into halloysite lumens did not change the paint color after one week of bright sunlight exposure, contrary to the sample prepared with loading unshelled silver nanoparticles. However, the yield of such silver-clay core-shell nanosystems was not high (ca. 10%), preventing the scaling up of the process; this is why checking this formulation for marine testing will be performed after the process optimization.

It is known that Mn^2+^ ions are also efficient against biofouling and have low toxicity. A natural mixture of MnO and halloysite could be used as an additive to the epoxy coating. MnO submicron particles associated with halloysite nanotubes were kindly provided to us by Northstar Mines LLC (Eureka, UT, USA). The EDS spectra clearly showed the presence of manganese ca. 300 nm in size at 8 wt.%. Traces of Mn ions were also distributed in the framework of the halloysite nanotubes. This mixture of clay with the micro/nano scale metal oxides is promising for the production of biocide additives to paint using such abundant natural materials. The antifouling performance of these materials was tested in the northern White Sea in July in a temperature range of 5–10 °C over 2 weeks. The test was performed in 5 L bottles with White Sea water doped with the bacteria fertilizers under moderate sun irradiation in Arctic conditions. Epoxy coatings loaded with 5% of Ag-halloysite-APTES and MnO-halloysite natural mixture showed an efficient protection and no visible fouling (Supplementary Materials, Figure S1).

### 3.4. Antibacterial Test of Epoxy Resin/Halloysite Coating Formualtions

**Figure 7:**
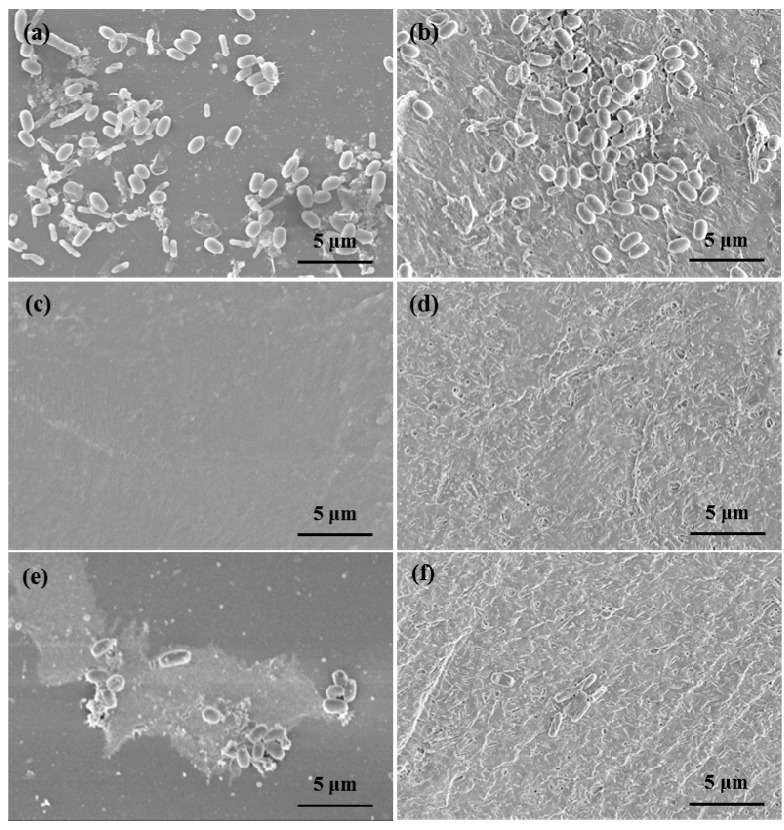
SEM images of the bacteria on the coating surface (left column—epoxy resin; right column—epoxy-halloysite formulations): (**a**) pure epoxy resin, (**c**,**e**) epoxy resin directly doped with DCOIT; (**b**) epoxy resin composited with pristine/empty halloysite, (**d**,**f**) epoxy resin with DCOIT-loaded halloysite. The samples were incubated in *Vibrio natriegens* suspension for 3 days (**c**,**d**) and after exposure to seawater for 60 days on a shaking platform (**e**,**f**).

After fixation with ethanol, *V. natriegens* proliferated on the composite surfaces were imaged with SEM to analyze the bacterial adhesion inhibition properties. The surface micrographs of the resin-HNT composite and the control samples incubated in the bacterial suspension (O.D._600 nm_ = 1.0) at 30 °C for 3 days were taken. Many bacteria were found on the surface of the pure epoxy resin (Figure 7a), as well as on the surface of control epoxy with empty halloysite (Figure 7b).

Total amount of DCOIT was the same (7.0 ± 0.5 wt.%) in both epoxy formulations with and without halloysite encapsulated. After 60 days of exposure to artificial seawater, the amount of bacteria proliferated on the surface of epoxy resin directly admixed with DCOIT increased significantly, and was estimated at ca. 4.0 × 10^8^/cm^2^ (averaged from 20 SEM images). There were very few bacteria on the surface of epoxy composites with halloysite encapsulated antifoulant (Figure 7d,f). The diffusion of antifoulant into the external marine environment was slowed down by the sustained release of DCOIT from halloysite, which prolonged its action over 2 months, leading to the significant improvement of the composite antifouling property.

**Figure 8:**
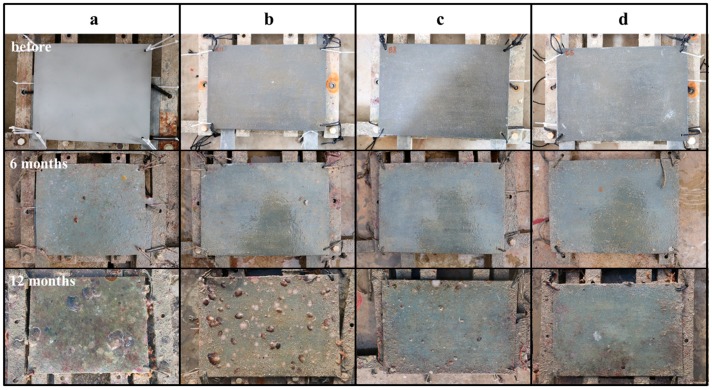
Photograghs of the flat panels uncoated (**a**); coated with epoxy resin directly doped with 5 wt.% DCOIT (**b**); and halloysite epoxy resin composits with 5 wt.% DCOIT plus 2 wt.% loaded in nanotubes (**c**); plus 5 wt. % loaded in nanotubes (**d**). Upper images—before; and lower images—after shallow submergence in the Sanya Bay, South China Sea, March 2018–February 2019.

This result was further confirmed by the field tests in Sanya Bay, South China Sea, Figure 8 (a—bare plate, b—coated with non-encapsulated DCOIT, and c,d—halloysite-encased antifoulant formulation). After six months, a 350 × 250 mm^2^ flat panel coated with resin composited with halloysite-encapsulated DCOIT remained clean, while marine microorganism colonies were observed on the uncoated control panel. After one year, the aggregates of microorganisms on the uncoated panel were larger than those on the non-encapsulated DCOIT epoxy, and their quantity/surface density was much smaller (Figure 8a–b). The epoxy resin doped with 5 wt.% of antifoulant without halloysite encapsulated showed fewer aggregates of microorganisms (Figure 8b).

Much less fouling was observed for HNTs-DCOIT-doped coatings: with 2 wt.% DCOIT after one year of exposure, one can see very few bacteria colonies (Figure 8c), and an increase in HNTs-DCOIT doping to 5 wt.% demonstrates a completely clean plate (Figure 8d), which is the best protection achieved. The halloysite formulations offered enhanced protection, which was especially spectacular after 12-month sea-immersion (right column). Halloysite antifoulant agent encapsulation drastically increases the protection property of epoxy resin composite with the optimal 9 wt.% DCOIT loaded halloysite doped at 22 wt.% in epoxy (~2 wt.% loading of encapsulated antifoulant) while preventing aggregation resulting with unwanted surface roughness increase.

## 4. Conclusions

Long-acting antifouling epoxy resin composites were prepared with 4,5-dichloro-2-octyl-isothiazolone (DCOIT) encased in halloysite clay serving as tubule nanocontainers for this chemical agent encapsulation and providing an extended release over one year. This nanocomposite formulation resulted in much longer protection as compared with DCOIT directly admixed into the epoxy paint.

The proliferation of *V. natriegens* on the halloysite-epoxy composite surfaces displayed good and long-lasting antifouling properties. As compared with the resin directly doped with non-encapsulated (fast-leaking) DCOIT, the halloysite nanoformulation gave resulted in much fewer microorganism aggregation spots being attached to the coating after exposure to seawater for 6–12 months. One-year marine antifouling panel tests in Sanya Bay, South China Sea showed only a few microorganism colonies attached on the sample coated with 2 wt.% DCOIT encapsulated in halloysite admixed to epoxy resin, while many microorganisms aggregated onto the coating doped with non-encapsulated DCOIT.

Such clay nanotube-fungicide-epoxy resin composites enabled long-lasting antimicrobial protection and could be used as coating materials for marine antifouling formulation. Halloysite is well dispersed not only in epoxy resin but also in most commercial paints, when tried (e.g., polyurethane and acrylic paints [40]). More than this, our halloysite nanocomposite formulation strategy is not restricted to this particular antifoulant, but may be extended to any other environmentally friendly antibacterial toxic agent, as well as to the surface “slippery” oil encapsulation, thus providing synergy of these two basic approaches in antifouling protection.

The antibacterial property of epoxy resin can also be improved with the addition of silver nanoparticles assembled on halloysite. This method may have an advantage for antifouling protection in cold Arctic waters, which is becoming much needed with the shift of marine petroleum exploration to northern seas.

## Supplementary Materials

The following are available online at https://www.mdpi.com/1996-1944/12/24/4195/s1, Figure S1: (**a**) TEM image and (**b**) EDS spectrum of natural mixture of MnO and halloysite.
