# Supplementary material for: Development of Marine Antifouling Epoxy Coating Enhanced with Clay Nanotubes
Source: Materials (Basel). 2019 Dec 13;12(24):4195. doi: 10.3390/ma12244195 (PMC6947195; doi:10.3390/ma12244195)
Supplement: Supplementary file 1 [file materials-12-04195-s001.pdf]

materials

Development of Marine Antifouling Epoxy Coating Enhanced with Clay Nanotubes

Ye Fu [1], Wencai Wang [2,*], Liqun Zhang [2,*], Vladimir Vinokurov [3], Anna Stavitskaya [3] and Yuri Lvov [3,4,*]

1. School of Materials Science and Mechanical Engineering, Beijing Technology and Business University, Beijing 100048, China; fuye@btbu.edu.cn
2. State Key Laboratory of Organic-Inorganic Composites, Beijing University of Chemical Technology, Beijing 100029, China
3. Department of Physical and Colloid Chemistry, Gubkin University, Moscow 119991, Russia; vladimir@vinokurov.me (V.V.); stavitsko@mail.ru (A.S.)
4. Institute for Micromanufacturing, Louisiana Tech University, Ruston 71272, LA, USA

* Correspondence: wangw@mail.buct.edu.cn (W.W.); zhanglq@mail.buct.edu.cn (L.Z.); ylvov@latech.edu (Y.L.)

Supplementary materials

Natural mixture of MnO and halloysite was provided to us from Northstar Mines Inc., (Grass Valley, CA, USA). The particle size of MnO was observed with TEM. The TEM image of MnO particle with the size of ca. 300 nm superimposed above the clay nanotubes is shown in Figure S1. The element analysis was produced with the EDS equipped TEM. The element amount of manganese is 8 wt.%.

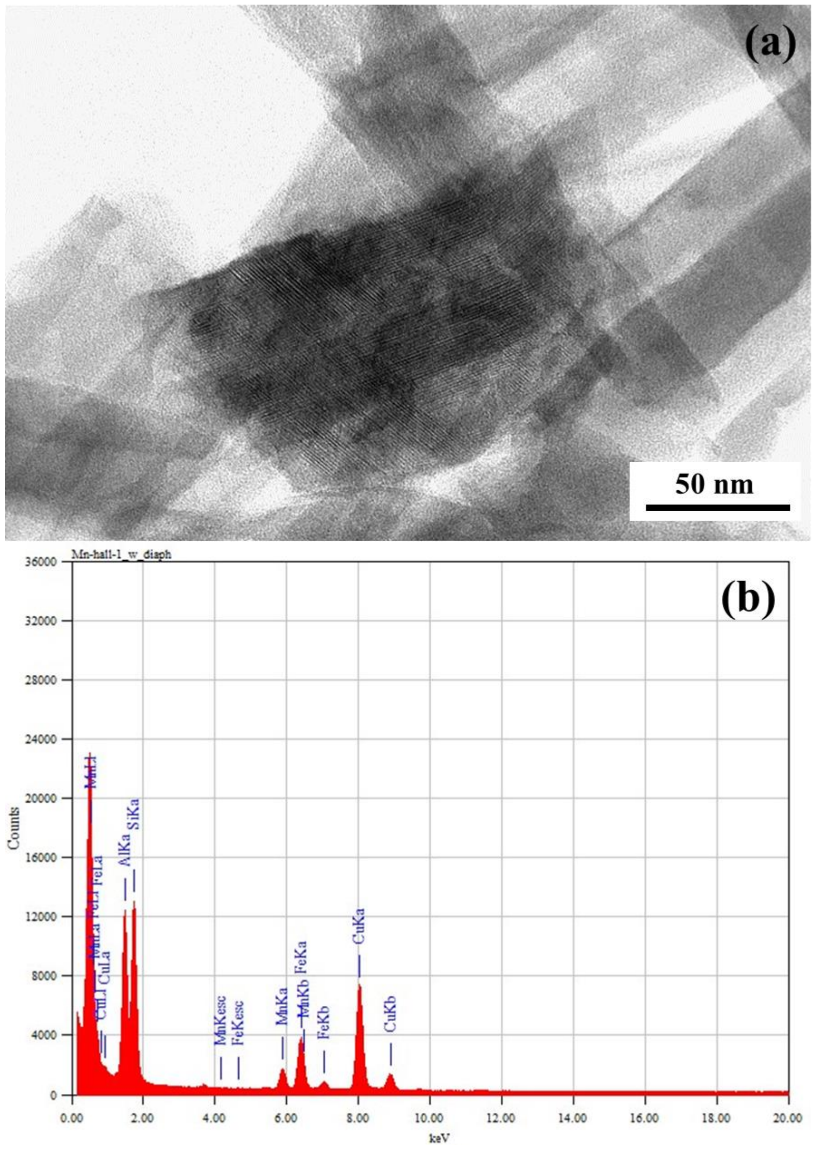

Figure S1. (**a**) TEM image and (**b**) EDS spectrum of natural mixture of MnO and halloysite.
